# Towards a sustainable set of European Core Health Indicators (ECHI)

**DOI:** 10.25646/12918

**Published:** 2024-12-11

**Authors:** Mariken J. Tijhuis, Eveline A. van der Wilk, Sarka Dankova, Angela Fehr, Silvia Ghirini, Mika Gissler, Romana Haneef, Heidi Lyshol, Emanuele Scafato, Stefanie Seeling, Hanna Tolonen, Thomas Ziese, Irisa Zīle-Velika, Peter W. Achterberg

**Affiliations:** 1 National Institute for Public Health and the Environment (RIVM), Centre for Public Health, Healthcare and Society, Bilthoven, The Netherlands; 2 Institute of Health Information and Statistics of the Czech Republic (UZIS ČR), Department of International Affairs, Prague, Czech Republic; 3 Robert Koch Institute, Centre for International Health Protection, Berlin, Germany; 4 Istituto Superiore di Sanità (ISS), National addiction and doping center, National Observatory for Alcohol (Osservatorio Nazionale Alcol - ONA), Rome, Italy; 5 Finnish Institute for Health and Welfare (THL), Helsinki, Finland; 6 Region Stockholm, Academic Primary Health Care Centre, Stockholm, Sweden; 7 Karolinska Institutet, Department of Molecular Medicine and Surgery, Stockholm, Sweden; 8 Santé Publique France (SPF), Department of Non-Communicable Diseases and Injuries, Saint-Maurice, France; 9 Norwegian Directorate of Health, Department of International Cooperation, Oslo, Norway; 10 Robert Koch Institute, Department of Epidemiology and Health Monitoring, Berlin, Germany; 11 Center for Disease Prevention and Control of Latvia (CDPC), Department of Research and Health Statistics, Rīga, Latvia

**Keywords:** Health Indicator, Public Health, European Union, ECHI, Health Information System, Metadata, Monitoring, Benchmarking, Health Promotion, Policy-making

## Abstract

**Background:**

The European Core Health Indicators (ECHI) are a set of 88 indicators that provide a compact overview of the extensive field of European public health and healthcare. The ECHI set adds value to European Union health information systems (HIS) for both Member States and EU-associated countries and the European Commission by providing a solid, comparable information base on national public health and healthcare trends and developments. The indicators allow for learning by comparison and the list supports the organisation of national health information systems. As the ECHI set was defined more than ten years ago, it is time to review its current needs and readiness for the future.

**Methods:**

In this article, we reflect on the sustainability of the ECHI set and explore directions for improving future use, based on the activities in the Joint Action on Health Information (2018 – 2021). There, we looked into ECHI governance and reviewed the set’s metadata, content and link with other indicator sets in the wider European health information landscape.

**Conclusions:**

We conclude that in order to remain relevant and keep up with technical and policy developments, the ECHI set needs maintenance and updates. This cannot be achieved in a non-systematic project-based manner; it requires sustainable funding, governance and formalised activities in a permanent structure. We call on the European Commission, Member States, research networks and individual users of the ECHI to take action in this.

## 1. Introduction

**Figure 1:**
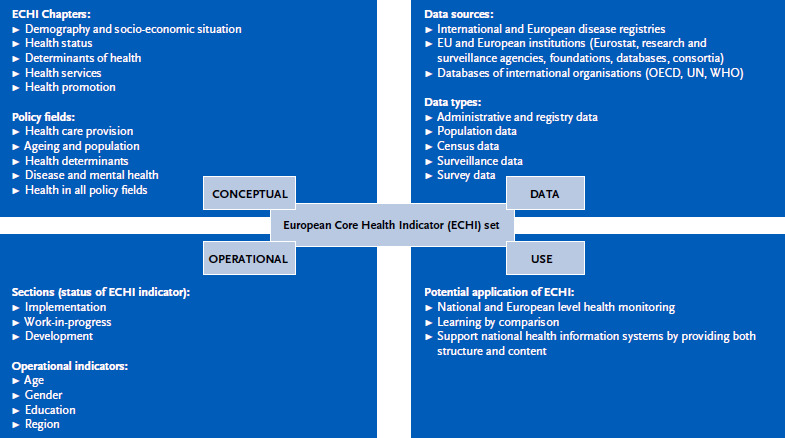
Main characteristics of the ECHI list. Source: adapted from Fehr et al. (2017) [4] OECD = Organisation for Economic Co-operation and Development, UN = United Nations, WHO = World Health Organization

**Figure 2:**
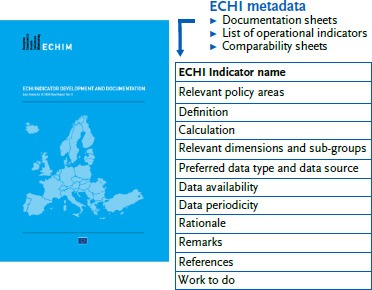
Front cover and main characteristics of the ECHI metadata as published in the Joint Action for European Community Health Indicators and Monitoring (ECHIM, 2009 – 2012). Source: adapted from Verschuuren et al. (2012) [3]

The European Core Health Indicators (ECHI) are a set of 88 indicators that provide a ‘snapshot’ of European public health and healthcare. The set adds value to European population health and healthcare systems by providing a compact, solid, comparable information base on public health and healthcare trends and developments in Member States (MS). The set is the result of European Commission (EC) co-funded MS efforts between 1998 and 2012, in the form of four consecutive projects [1]. This was initiated by an EC call to establish an indicator list as the core of the European Union (EU) public health monitoring system, following the adoption of a Community action programme on health monitoring [2]. Figure 1 provides an overview of the list’s main characteristics. The indicators cover five sections: ‘demographic and socio-economic’ (*n* = 9), ‘health status’ (*n* = 32), ‘health determinants’ (*n* = 14), ‘health services’ (*n* = 29) and ‘health promotion’ (*n* = 4). Some of the indicators are not yet implemented, i.e. they cannot be calculated (yet) in a comparable manner by most MS. For each indicator metadata are available [3], consisting of a documentation sheet, a list of relevant dimensions and a comparability sheet [3], see Figure 2. ECHI documentation sheets contain the technical information needed for computing the indicator as well as contextual information required for interpreting the indicator, in a structured format delivered by the ‘Joint Action for ECHIM’ (European Community Health Indicators Monitoring). The list of relevant dimensions provides a quick summary per indicator of the definitions and breakdowns required for the indicator. The comparability sheets provide an overview of the main comparability or quality issues for the indicator, important for the interpretation of the indicator.

The ECHI indicators and their metadata can be accessed through the ECHI data tool [5], an interactive application which is maintained by the Directorate-General (DG) Health and Food Safety (SANTE) in collaboration with DG Eurostat – European Statistics (EUROSTAT). The ECHI do not have legal status, but through EC regulations [6–12], MS are obliged to produce at least part of the statistical data that are needed to calculate the indicators. This increases data availability. Also beneficial in this respect is collaboration with non-EU international entities, e.g. the World Health Organization’s Regional Office for Europe (WHO/Europe) and the Organisation for Economic Co-operation and Development (OECD).

**Key statements**

► The ECHI set adds value to EU health information systems by providing a solid, comparable information base on national public health and healthcare trends and developments.
► It is essential to institute ECHI governance. In this, we see a role for representatives from the European Commission, from Member States and from international organisations.
► The European Commission is seen as important partner to Member States, with a role in securing ECHI policy relevance, technical commitment, financial sustainability and possibly legal status.
► The ECHI metadata and the content and suitability of the ECHI set need to be reviewed regularly, e.g. every three years.
► Visibility of the ECHI set is a prerequisite for its use – some options are listed for improving communication and increasing visibility for the European Commission and Member States.

Since 2006, the ECHI have been an underlying drive for EU-wide data collections, such as the European Health Interview Survey (EHIS) [13] or the EU Statistics on Income and Living Conditions (EU-SILC) [14], to fulfil policy needs. In addition, they have fed the discussion for complementing self-reported data with European health examination data for major public health problems that are difficult to measure objectively, such as overweight or high blood pressure [15]. The ECHI indicators are important building blocks for the ‘EU State of Health Country Profiles’ and ‘Health at a Glance: Europe’, reports aiming to make health system information, expertise and best practices easily accessible to policy-makers [16]. The ECHI set allows for learning by comparison and can support national health information systems by providing both structure and content.

The last ECHI project ended in 2012. Since then, some evaluations have been carried out [17–19]. The Public Health Evaluation and Impact Assessment Consortium (PHEIAC) [17] concluded more than ten years ago that the combination of financial constraints and poor visibility/recognition in the formal policy-making process restricts the support for ECHI and that the ECHI would benefit from a clearer legal status. They advised to address financing issues, both for individual indicators and for having the ECHI system in place. The EC cannot finance permanent or frequently recurring costs for the same activity through project funding and this has long been known to hamper sustainability. In 2018, the BRIDGE Health Project (BRidging Information and Data Generation for Evidence-based Health policy and research) noted that a mechanism to officially update the set’s content and metadata is still lacking [19]. This poses the important question, especially in a world that is rapidly evolving, whether the set is future-proof.

In this article, we review the sustainability of the ECHI set and we aim to provide suggestions and recommendations that will benefit and improve its future use. The evaluation is based on work conducted within the Joint Action on Health Information (InfAct, 2018 – 2021), whose overall aim it was to build a sustainable infrastructure for EU health information [20]. The results on ECHI can be found summarised in a detailed report available from the InfAct website [21] as well as from the European Health Information Portal (HIP) [22]. The HIP was set up as a one-stop shop facilitating access to population health and healthcare data, information and expertise in Europe [23], and the concept of National Nodes on Health Information (NNHI) was introduced, as organisational entity, often linked to a national institution or governmental unit that functions as a national liaison and brings together relevant national stakeholders in EU Member States in a systematic way [24]. These elements have been further developed in the Population Health Information Research Infrastructure (PHIRI) project [25] and are further described on the HIP. These initiatives fit in the ongoing work towards a sustainable infrastructure for European Health Information [26].

## 2. Considerations for the ECHI set

Important considerations for an indicator set are who is responsible for maintaining the set (its governance), whether the set still covers MS/EC needs (relevance), whether the metadata are still up-to-date, and whether the set is found and taken up in practice (visibility and use). We discuss these aspects below.

### 2.1 Governance and tasks

Maintaining an indicator set involves several tasks. For the ECHI support process, we identify the following:

► Carrying out update procedures. This includes metadata maintenance, proposing changes in indicator definitions, preferred data sources and/or new indicators, making use of existing comparable indicators where possible, implementing input from OECD, WHO/Europe, EU institutions and research networks
► Carrying out regular evaluations of (MS and other) user needs and meeting them
► Maintaining and further developing the data presentation tool (ECHI tool)
► Promoting the use and visibility of ECHI
► Supporting MS in ECHI implementation
► Increasing collaboration with international organisations and across the EC, aligning with international frameworks

Currently, governance or updating procedures for the ECHI are not formalised, i.e. no entity carries the formal mandate to deliver updated ECHI metadata and ensures that the set fits policy needs. This puts previous efforts at risk. The ECHI set could become a product for which a need was identified and was successfully developed by MS on a project basis. But that is not kept up-to-date, has no infrastructure to sustain it and thereby loses value. Thus, it is essential to institute ECHI governance. In this, representatives from the EC, from MS and from international organisations will need to fulfil a role. Different roles and models have been discussed within InfAct, the below reflections thus represent some of several possibilities.

**Figure 3:**
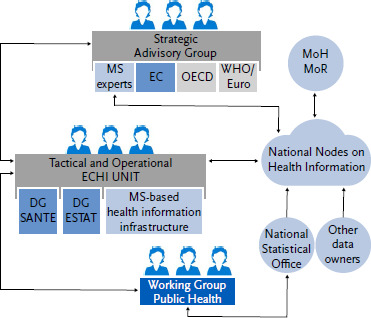
Proposed elements for ECHI governance. Source: adapted from Tijhuis et al. (2021) [21] MS = Member States, EC = European Commission, OECD = Organisation for Economic Co-operation and Development, WHO/Europe = World Health Organization Regional Office for Europe, DG SANTE = Directorate General Health and Food Safety, DG ESTAT = Directorate General Statistical Authority of the European Union (Eurostat), MoH = Ministry of Health, MoR = Ministry of Research; the National Statistical Offices, the Working Group Public Health and Eurostat are part of the European Statistical System. See text and Infobox 1 for further explanation.

Suggested elements of ECHI governance are: an ECHI unit at EU-level (the ‘secretariat’), a Strategic Advisory Group (SAG), National Nodes on Health Information (NNHI), the Working Group on Public Health Statistics (WG-PH) [27], national statistical offices, other data owners and Ministries of Health and Ministries of Research, see Figure 3 and Infobox 1 for a visualisation and a summary in text, respectively. The ECHI unit would consist of health information experts with ECHI knowledge from MS as well as from DG EUROSTAT and DG SANTE, covering public health, health services, epidemiology, health statistics, data handling and information communications technology (ICT). In this unit, DG EUROSTAT and DG SANTE would be invaluable in connecting the ECHI process with developments in European statistics and policies as well as creating practical, financial and legislative space for the ECHI process. We recommend that the EC considers a direct agreement or service contract with an EU-structure and supports its legal status for a longer time period (e.g., a minimum of ten years) and gives it a clear mission and governance structure. In addition, strong MS commitment is also essential. Possibly, the establishment of national Health Data Access Bodies (HDABs) in response to the European Health Data Space (EHDS) Regulation [28], will contribute to facilitated governance and completeness of ECHI, via more FAIR and higher quality data.

**Infobox 1**

**Suggested core elements of ECHI governance.** Source: adapted from Tijhuis et al. (2021) [21]

ECHI unit

► This unit consists of health information experts with ECHI knowledge - the ‘secretariat’:
  ► DG ESTAT: information and communications technology (ICT) expert, statistician
  ► DG SANTE: coordinator, ICT expert, health information expert
  ► National health information experts affiliated with a sustainable health information research infrastructure
► Main task: carry out the ECHI tasks (see text)
► The members of the unit are in informal contact as much as necessary

Strategic Advisory Group

► This group consists of national health information experts and representatives from OECD, WHO, relevant EU institutions, research networks, others. A natural starting point could be the EU Expert Group on Health Information (EGHI, if re-instated) and the Expert Group on Health Systems Performance Assessment (EG-HSPA)
► Main task: provide recommendations to the ECHI unit (based on input by the ECHI unit or of its own accord), function as steering group

National Nodes on Health Information (NNHI)

► The NNHI will deliver one primary and one secondary contact person for each MS
► Main task: knowledge brokering regarding the data and information situation within countries, i.e. discuss with national Ministries and all relevant actors; set up National Implementation Teams to facilitate national implementation

Working Group on Public Health (WG-PH) at EUROSTAT

► Main task: deliver statistical approval (‘ECHI stamp’)
► Take up in regular meeting at least once a year

National Statistical Offices and other relevant data owners

► Via WG-PH and NNHI
► Main task: deliver the necessary national statistical and health information knowledge; Coordinate and improve data exchange procedures and mechanisms

Ministries of Health and Ministries of Research

► Via NNHI, Public Health Expert Group (PHEG), EGHI and EG-HSPA
► Main task: as main national users, contribute user needs and validate changes to the ECHI list

National Statistical Offices, National Public Health Institutes and Ministries of Health can support the process with expert national input and in-kind commitment through the Working Group on Public Health (WG-PH) [27], the Expert Group on Health Information (EGHI, if re-instated), the Expert Group on Health Systems Performance Assessment (EG-HSPA), the Public Health Expert Group (PHEG) and National Nodes on Health Information (NNHI) proposed by InfAct and further developed by PHIRI. European research networks and institutions could contribute their valuable expertise, such as the European Centre for Disease Prevention and Control (ECDC), the Union Drugs Agency (EUDA) and the Joint Research Centre (JRC). A Strategic Advisory Group including members from non-EU public health institutions in the European Region, such as WHO/Europe and the OECD, is also essential, to keep up with developments in the Region, to ensure the best available data is used and to reduce reporting burden.

### 2.2 A relevant and up-to-date set of indicators

The ECHI indicators were developed to be a consistent and balanced set, covering all relevant public health areas. However, health information needs and health information systems, including data sources and data collection methods, change. Consequently, monitoring tools need to adapt and develop with it. This was already highlighted in the early development of the ECHI set. Criteria for adding and deleting indicators were developed [3], the set was designed to be a flexible system that could reflect new developments in public health issues. We reviewed these criteria within InfAct [21], see Infobox 2 and Infobox 3, and suggest they be applied in the content evaluation and update procedure, after formalisation by the new ECHI unit.

The content and suitability of the ECHI set need to be reviewed regularly, using an appropriate prioritisation process. A Delphi procedure, including experts and stakeholders, could be considered as a feasible method for a regular review [29, 30]. Within a European permanent governance structure, such an exercise could be based on a core panel of public health experts to be complemented by topic-specific expertise for review of specific indicators. A major advantage of the ECHI set as compared to other initiatives developing indicators sets, is that the major players in the field of health information (i.e., EC DG SANTE/EUROSTAT, WHO/Europe, OECD, MS via their health information experts) were (already) involved in its development. This is a good basis to set up an active and engaging Delphi process. In the process, due attention needs to be given to indicators that are no longer considered relevant (using the criteria for deletion), to prevent overgrowth; more reflections on (optimal) size are given elsewhere [19].

It is important to be aware of other indicator sets in the European Region and discover where they differ meaningfully and where harmonisation could take place. We think of initiatives taking place at OECD and WHO/Europe, but also within the EU, such as the Joint Assessment Framework in the area of Health (JAF Health) indicator list [31], developed in 2013 with the support of the EC services (in particular DG Employment, Social Affairs & Inclusion (EMPL) and EU-ROSTAT, with consultation of DG SANTE and DG for Economic and Financial Affairs (ECFIN)). We identified differences and commonalities with JAF Health through a comparison of both indicator sets over several dimensions [21]. These indicator sets were prepared for different purposes, but could serve as inspiration for each other’s improvement and should be harmonised in areas which they have in common. An important overarching set of goals and targets are the Sustainable Development Goals (SDGs) [32]. In the European Region, these are being monitored by individual MS, EC, WHO/Europe and OECD, each with their own specific indicators and operationalisations. A clearer link between the ECHI and the SDGs is recommended, both from a perspective of reporting burden and longer-term monitoring. More recent initiatives in the field of health determinants worthwhile monitoring for relevant crosslinkage are the Joint Action PreventNCD (https://preventncd.eu/) and the Joint Action Cardiovascular Diseases and Diabetes – JACARDI (https://jacardi.eu/).

**Infobox 2**

**Criteria for the selection of ECHI shortlist indicators (stable set)**

Source: adapted from Tijhuis et al. (2021) [21]

- The list should cover the entire public health field, including health system performance, following the commonly applied structure of the well-known Lalonde model: health status, determinants of health, health interventions/health services, and socio-economic and demographic factors.
- The indicators should serve the user’s needs, meaning that they should support potential policy action, both at the EU and Member State level.
- Criteria should be applied to identify and prioritize needed policy action (e.g. large public health problems, including health inequalities) and best possibilities for effective policy action.
- Existing indicator systems, such as the indicators used in the WHO Health for All database and OECD Health Data, should be used as much as possible, but there is room for innovation.
- Focus on the best possibilities for effective policy action.

**Infobox 3**

**Criteria for additions and deletions to the ECHI shortlist indicators (stable set)**

Source: adapted from Tijhuis et al. (2021) [21].

Criteria for additions

- The indicator should have clear policy relevance, i.e. it should be related to a major public health issue in Europe. A major public health issue is a policy relevant issue when it has a high burden of disease, clear possibilities or needs for prevention, and/or clear possibilities for reducing health inequalities (is actionable). The importance of the issue shall be determined by adequate prioritization and evidence-based public health methods, involving stakeholder expertise.
- The indicator should not disturb the balance of the ECHI shortlist, i.e. there should not be too many (overlapping) indicators for similar topics, and not too many indicators for ‘minor’ or contextual topics in the shortlist.
- The shortlist should provide a ‘snapshot’ of public health, covering determinants, status, services and contextual information, from the point of view of the public health generalist.
- The indicators in the shortlist should be suitable for providing a benchmark in terms of reflecting time trends.
- The indicators in the shortlist should be suitable for providing a benchmark in terms of international (EU) comparisons, i.e. should be accessible and comparable across countries.
- The indicator is expected to be relevant for a longer period of time.
- Disaggregations should be available for the indicator (age, sex, geography).

Criteria for deletions

- The indicator is related to a topic that is no longer policy relevant.
- A new and better indicator has been identified for the same concept.
- There is lack of between-country differences.

Next to an update based on policy relevance, a procedure is needed to keep the metadata valid and up to date. Notably, reviewing the ECHI metadata is a very time-consuming process, which requires adequate and competent human resources and efficient organisation. In InfAct, researchers from different institutes (Robert Koch Institute, Germany; Institute of Hygiene, Lithuania; Institute of Health Information and Statistics of the Czech Republic, Czech Republic) conducted a review of the documentation sheets as available from the 2012 ECHI report [3]. Following a pilot review, each of the three institutes reviewed the documentation sheets independently. Revisions to the documentation sheets were tracked, the reviewers compared results, resolved disagreements and compiled them into final peer-review draft documentation sheets. Points for discussion were collected in separate tables. The review was shared with EC (DG SANTE and EUROSTAT) health information experts to have their inputs. A new ECHI unit may start from the remarks and recommendations that were collected [21], i.e. tabulated discussion points and draft revised documentation sheets. The metadata require updating not only in terms of the information that is collected but also in terms of the method of dissemination. We suggest making all ECHI metadata and data officially available to the health information community in digital format and establishing a digital system for suggestions on ECHI documentation sheets and on ECHI topics to make full use of the international health information community. A source of inspiration could be the ICD-11 (International Classification of Diseases for Mortality and Morbidity Statistics) platform, which allows registered users to contribute to ICD-11 development by commenting, suggesting changes or enhancing content [33]. We suggest that a combined content and metadata updating procedure is carried out every three years.

In the update process, it is essential that clarity and perspective are also provided on the indicators that are not implemented yet; this refers to the ‘work-in-progress section’ (i.e., indicators technically nearly ready for incorporation in regular international data collections) and the ‘development section’ (indicator concepts not yet ready for incorporation in international regular data collections). The work-in-progress and development indicators have deliberately been kept as part of the ECHI set as not to lose sight of them, but of course they were meant to progress to the ‘implemented section’. A separate working group could be set up for the work-in-progress and development indicators. This group could set clear targets regarding the timeframe for their development and work together with other initiatives dealing with similar development issues, e.g., the WHO/Europe development list in the European Program of Work Measurement Framework.

**Infobox 4**

**Criteria for additions and deletions to a flexible and actionable subset of ECHI shortlist indicators**

Source: adapted from Tijhuis et al. (2021) [21]

Criteria for additions

- The indicator is relevant for an emerging problem in public health.
- The indicator is suitable for providing a benchmark for international (EU) comparisons.

Criteria for deletions

- The indicator is no longer comparable, used or considered sufficiently relevant by experts.
- The indicator requires transfer to the stable set.

Stable indicators are important to provide insight in trends, but may not always be compatible with the need for actionable information on emerging issues. Health issues may arise that require swift attention and comparisons across EU MS that a stable ECHI set cannot accommodate. The recent COVID-19 experience highlights this need. For these cases, we propose to establish, next to a stable core set, a more flexible subset in the ECHI set that could address this need under the flag of ECHI (see Infobox 4 for proposed criteria). The concept was supported by health information experts already during the BRIDGE Health project (2015 – 2018) [19].

### 2.3 Visibility and use

ECHI still has not gained a place in the formal policy-making process, overcome financial constraints or gained legal status, as was concluded earlier [17]. The information about the ECHI that is currently available on the EC website [34] is limited and previous websites from the ECHI projects no longer exist. We collected historic and practical information on the ECHI set and disseminated this via the European Health Information Portal (HIP), as a central knowledge repository where interested users can find more information about ECHI [23]. This includes reports from the ECHI projects, evaluations, and examples of use of the ECHI by MS and in international reports.

The value and the use of the ECHI could be promoted through capacity building activities in curriculums for Public Health training, e.g. by the Association of Schools of Public Health in the European Region (ASPHER) or via the HIP which provides an overview of past and upcoming health information related trainings. In addition, it may be considered to routinely address ECHI in EU Parliament and to prepare an ECHI website specifically for policy-makers to consult as a primary source of reliable information in policy dialogues and in discussing health related issues. Evidence-informed policy-making requires data speaking to and being heard by policy-makers. This requires an information system that also includes e.g. knowledge translation and the right infrastructural conditions.

**Figure 4:**
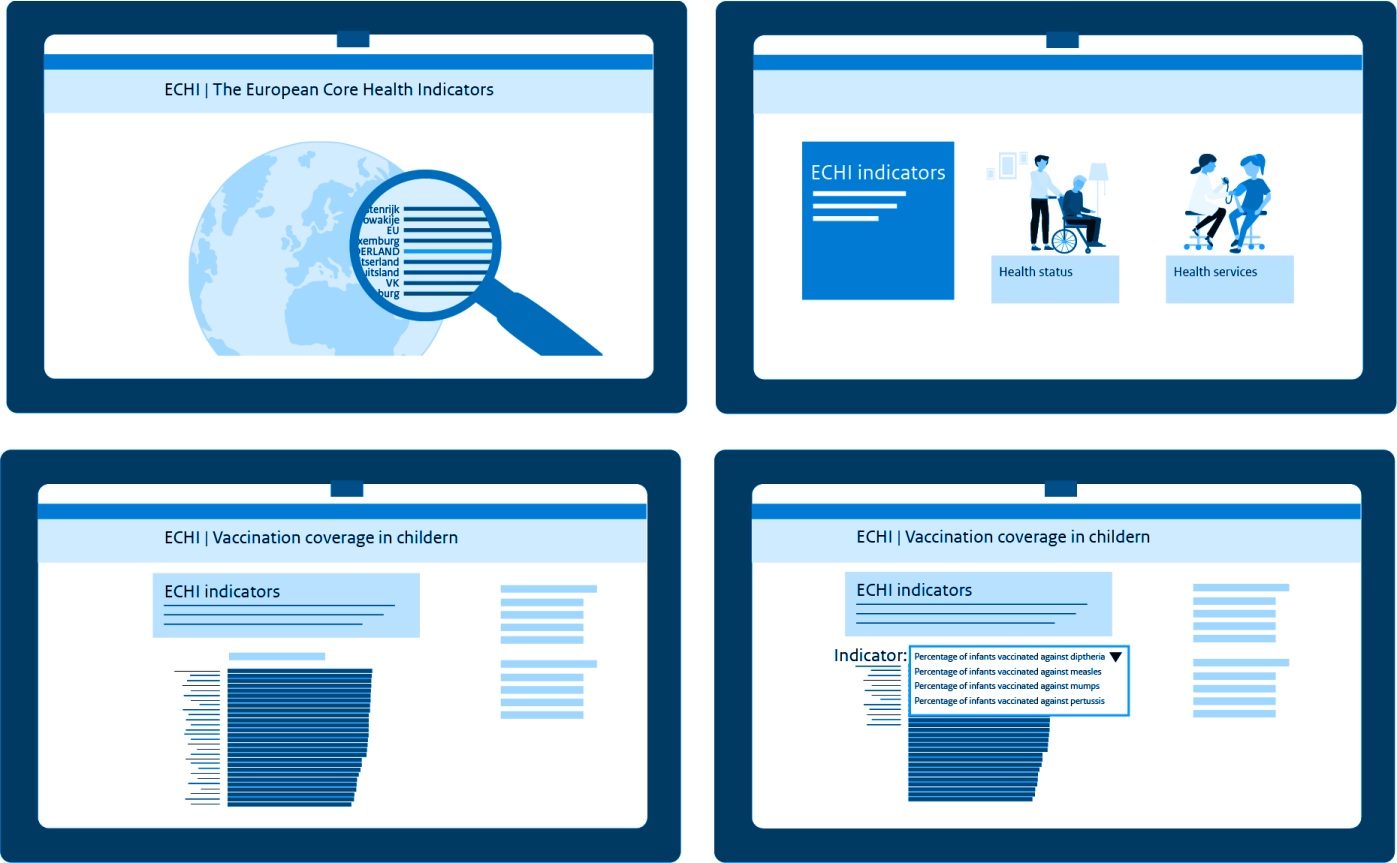
Simplified screenshots of ECHI indicators on the Dutch website VZinfo.nl, taking ECHI indicator ‘Vaccination coverage in children’ as an example. Source: adapted from National Institute for Public Health and the Environment, RIVM (2024) [35]

Visibility of the set is a prerequisite for its use. Here we list some options for improving communication and increasing visibility for EC and MS. They could set up a social media account to point at relevant developments in the field of Public Health in Europe related to ECHI and exchange ideas on how to make use of ECHI (adding tags like #ECHI-indicator). This could be linked to the updates that are currently already given by EUROSTAT in the ECHI data tool [5], which we recommend to be further expanded. ECHI outcomes could be visualised more often by means of infographics. Furthermore, ECHI ambassadors could be appointed and given an online platform. Other suggestions are to organise symposia to exchange experiences with the use of ECHI, implement ECHI into national data and presentation tools, invite countries to make a national web page dedicated to ECHI (this can be basic, with a possible link to the ECHI tool) and to involve National Nodes on Health Information as much as possible. See Figure 4 for a schematic example of a national website on public health and healthcare information displaying an ECHI indicator (www.vzinfo.nl).

## 3. Conclusions

The continued availability and analysis of good and timely data that are comparable over time and between countries, presented in a clear and understandable manner, are of value to both the EU and the individual MS, to support evidence informed health policies. In these times of seemingly endless data potential and excellent initiatives driven by the EHDS regulation, we must not forget that specific processing and knowledge are required to be able to interpret and learn from the data. In other words: we must not forget to take care of the full scope of our health information systems. The ECHI set can support this function and thereby add value to European population health and MS health information systems, and strengthen evidence-informed policies and actions. In order to remain relevant and keep up with technical and policy relevant requirements, it needs maintenance and further development. This cannot be achieved in a non-systematic project-based manner and requires sustainable action.

First and foremost, a sustainable governance structure is needed, as the ECHI set lacks an official and functioning mechanism to maintain and improve it. We see the EC (DG SANTE and DG EUROSTAT in particular) as important partners of MS, with an urgent role in promoting policy relevance and contributing technical commitment, financial sustainability and possibly legal status for the ECHI. In addition, it remains important to strive for synergy and alignment with OECD and WHO/Europe. Both the policy relevance and the metadata need to be reviewed regularly (e.g., every three years) and the metadata need to be disseminated in an easily accessible way.

We call on the EC, MS, Research Networks and individual research- or policy-based users of the ECHI indicator set to act and contribute to this in the following ways: MS via active participation, e.g., via National Nodes on Health Information; Research Networks via claiming and fulfilling an active role in developing and maintaining indicators in their knowledge domain; Users via contributing to the debate on the ECHI. Most importantly, we call upon the EC to create the right environment for ECHI governance, supporting a financial and legal framework and providing the expertise to fill the ECHI tool and expand its functionalities.
